# Structural, microstructural, magnetic and electromagnetic absorption properties of spiraled multiwalled carbon nanotubes/barium hexaferrite (MWCNTs/BaFe_12_O_19_) hybrid

**DOI:** 10.1038/s41598-021-95332-9

**Published:** 2021-08-05

**Authors:** Nurshahiera Rosdi, Raba’ah Syahidah Azis, Ismayadi Ismail, Nurhidayaty Mokhtar, Muhammad Misbah Muhammad Zulkimi, Muhammad Syazwan Mustaffa

**Affiliations:** 1 Institute of Advanced Materials, Universiti Putra Malaysia, 43400 UPM Serdang, Selangor, Malaysia; 2 Department of Physics, Faculty of Science, Universiti Putra Malaysia, 43400 UPM Serdang, Selangor Malaysia

**Keywords:** Materials science, Nanoscience and technology, Physics

## Abstract

Microwave absorption properties were systematically studied for synthesised barium hexaferrite (BaFe_12_O_19_) nanoparticles and spiraled multiwalled carbon nanotubes (MWCNTs) hybrid. BaFe_12_O_19_ nanoparticles were synthesised by a high energy ball milling (HEBM) followed by sintering at 1400 °C and structural, electromagnetic and microwave characteristics have been scrutinized thoroughly. The sintered powders were then used as a catalyst to synthesise spiraled MWCNTs/BaFe_12_O_19_ hybrid via the chemical vapour deposition (CVD) process. The materials were then incorporated into epoxy resin to fabricate single-layer composite structures with a thickness of 2 mm. The composite of BaFe_12_O_19_ nanoparticles showed a minimum reflection loss is − 3.58 dB and no has an absorption bandwidth while the spiraled MWCNTs/BaFe_12_O_19_ hybrid showed the highest microwave absorption of more than 99.9%, with a minimum reflection loss of − 43.99 dB and an absorption bandwidth of 2.56 GHz. This indicates that spiraled MWCNTs/BaFe_12_O_19_ hybrid is a potential microwave absorber for microwave applications in X and Ku bands.

## Introduction

Microwave is an electromagnetic (EM) radiation with a frequency range between 300 MHz to 3 THz. The increasingly growing use of electronic devices operating at microwave frequencies has resulted in the increase of electromagnetic (EM) interference and non-ionising radiation. This leads to the increased demand of microwave absorbing materials to reduce interference, shield devices, regulate shield rooms and chambers for EM compatibility and reduce the harmful effects of EM waves on biological tissues. For example, in the field of defense (military)^1,2^, this microwave absorber is used as the coating or painting of defense equipment and installations such as stealth aircrafts, warships and military uniforms, particularly for the guards. The microwave absorbing materials (MAMs) can be categorised into two: magnetic and dielectric absorbing materials. Excellent MAMs should be lightweight, thin, wide-coverage, absorbent and simple (having simple coating-layer structure). The material's conductivity, dielectric permittivity and magnetic permeability could affect absorption loss. As a result, heat loss due to the interaction of the EM field with the electrical and/or magnetic dipole causes absorption loss in the material. In this case, magnetic (″) and dielectric (″) losses are accounted for most absorptions.

M-Type barium hexaferrite (BaFe_12_O_19_) forms SRS*R* crystal structure, in which R and S indicate three and two oxygen-ion layer blocks. These ferrites are effective microwave materials due to their high magneto-crystalline anisotropy, low cost, high Curie temperature, and competent saturation magnetization properties^3^. Meanwhile, multiwalled carbon nanotubes (MWCNTs) are the most widely researched materials for radar absorbing applications because of their specific characteristics such as high conductivity, elevated aspect ratio and super mechanical strength^4–6^. CNTs composites have many advantages due to their ability to adapt to the dielectric properties and to possess lightweight structures without reducing the mechanical properties. Based on the literature, many researchers reported on the preparation of CNTs mixture with ferrite^7–9^. In the present research work, spiraled MWCNTs/BaFe_12_O_19_ hybrid will be synthesised via CVD process by using BaFe_12_O_19_ sintered powder as a catalyst. MWCNTs/BaFe_12_O_19_ will be attached to each other, and this will increase the energy transfer from one medium to another medium. Hypothetically, the EM wave absorption becomes more efficient. The spiraled MWCNTs/BaFe_12_O_19_ hybrid will be prepared to investigate its performance on the EM wave absorption with a wideband frequency capability. The spiraled MWCNTs/BaFe_12_O_19_ hybrid may be used as a potential microwave absorber as well as a protective coating.

## Experimental procedure

### Preparation of BaFe_12_O_19_

The details of extraction and purification of Iron oxide (Fe_2_O_3_) from mill scale waste have been reported elsewhere^10^. Fe_2_O_3_ obtained by oxidation process from mill scale waste was mixed and weighed with barium carbonate (BaCO_3_) (99.8%, Alfa Aesar) according to stoichiometry formula. Then it underwent a high energy ball milling with 10:1 ball to powder ratio (BPR) using a SPEX8000D HEBM milling machine for 3 h. Then the milled powders were sintered at 1400 °C for 6 h in an ambient atmosphere**.**

### Preparation of spiraled MWCNTs/BaFe_12_O_19_ hybrid

**Figure 1:**
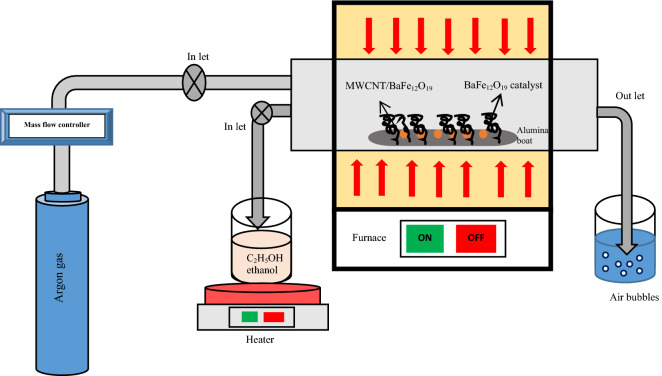
Schematic diagram of a chemical vapour deposition (CVD) set up in its simplest form.

The sintered powder of BaFe_12_O_19_ was used as a catalyst and ethanol solution (C_2_H_5_OH) (96%, Sigma Aldrich) as a carbon source to synthesis spiraled MWCNTs via chemical vapour deposition (CVD) method (Fig. 1). 1.0 g of BaFe_12_O_19_ was inserted into the middle of the furnace with an argon flow of 100 sccm. As the furnace reached the targeted synthesis temperature of 750 °C, the evaporated ethanol solution at 100 °C temperature was flowed in for 30 min. Then the furnace was left to cool down to room temperature under an argon environment before the sample was taken out for further analysis. The synthesised temperature using CVD was at 750 °C while the temperature of BaFe_12_O_19_ phase formation was set at 1400 °C. Hence, when low temperature was used to synthesise CNTs as compared to the high temperature of BaFe_12_O_19_, there were no phase changes detected.

### Preparation of spiraled MWCNTs/BaFe_12_O_19_ hybrid/epoxy

The composite was produced by mixing spiraled MWCNTs/BaFe_12_O_19_ hybrid powders with an epoxy resin (Araldite 506, Sigma Aldrich) in a 2 wt%, 4 wt%, 6 wt%, 8 wt% and 10 wt% of filler content. The mixture was poured into a different rectangular-shaped sample holder (model WR 90) of 2 mm thickness and left dried overnight at room temperature.

### Characterisations

The phase identification was determined using X-Ray Diffraction (XRD) (Philips X’pert Diffractometer model 7602 EA Almelo) with CuKα radiation that was set at a wavelength of λ = 1.5406 Å. The surface morphology was observed using the Field Emission Scanning Electron Microscopy (FESEM) (FEI Nova NanoSEM 230) and the High-Resolution Transmission Electron Microscopy (HRTEM) (LEO 912AB Energy Filter). The elemental composition of the samples was detected with an Energy-Dispersive X-ray (EDX) (Oxford Instruments) system. The determination of the molecular structure and the degree of graphitisation of the CNTs were studied using Raman spectroscopy (WITec Raman spectrometer model Alpha 300R). The measurement of electromagnetic EM wave properties of spiraled MWCNTs/BaFe_12_O_19_ hybrid was performed using a Vector Network Analyzer (VNA) (PNA N5227A) in the frequency range of 8–18 GHz.

## Results and discussion

### Phase and microstructural analysis

**Figure 2:**
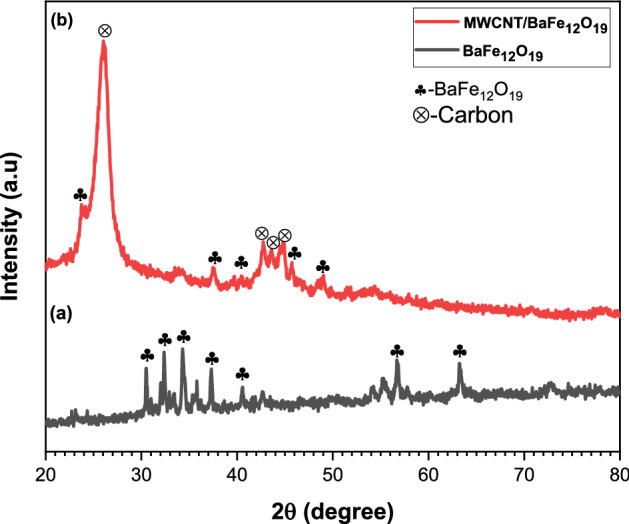
XRD pattern of (**a**) barium hexaferrite (BaFe_12_O_19_), (**b**) spiraled MWCNTs/BaFe_12_O_19_ hybrid via CVD.

X-ray diffraction patterns of both BaFe_12_O_19_ and spiraled MWCNTs/BaFe_12_O_19_ hybrid were detected (Fig. 2), and all peaks were identically indexed to BaFe_12_O_19_ with no impurity phases detected based on the ICSD card no. 98-004-7018, (Fig. 2a). The prominent peaks of BaFe_12_O_19_ were located at 2θ = 30.48°, 32.37°, 34.31°, 37.32°, 56.69° and 63.25°. The XRD spectra of spiraled MWCNTs/BaFe_12_O_19_ hybrid was ascribable to the MWCNTs carbon peaks and the BaFe_12_O_19_ patterns (Fig. 2b). The diffraction peak appeared at 2θ = 25.9° with an *hkl* index of (002) corresponded to the MWCNTs structure^11^ and other carbon peaks located at 2θ = 42.73°, 43.45° and 44.81°. However, there were some traces of BaFe_12_O_19_ detected due to the low amount of BaFe_12_O_19_ which was about 1 g used as the catalyst. The signal of BaFe_12_O_19_ was suppressed by a higher signal of MWCNT (Fig. 2b). The existence of MWCNTs peaks confirmed the successful synthesis of spiraled MWCNTs/BaFe_12_O_19_ hybrid.

**Figure 3:**
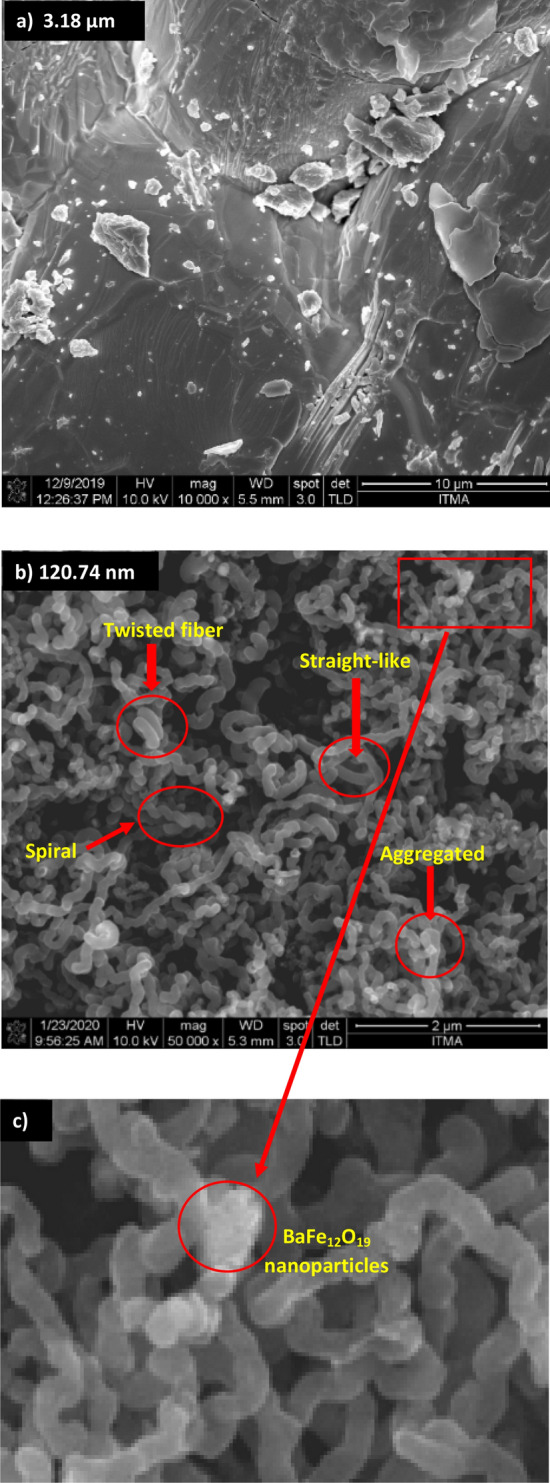
FESEM images of (**a**) barium hexaferrite (BaFe_12_O_19_), (**b**) FESEM images of spiraled MWCNTs/BaFe_12_O_19_ hybrid, (**c**) enlarge image of BaFe_12_O_19_ nanoparticles.

**Figure 4:**
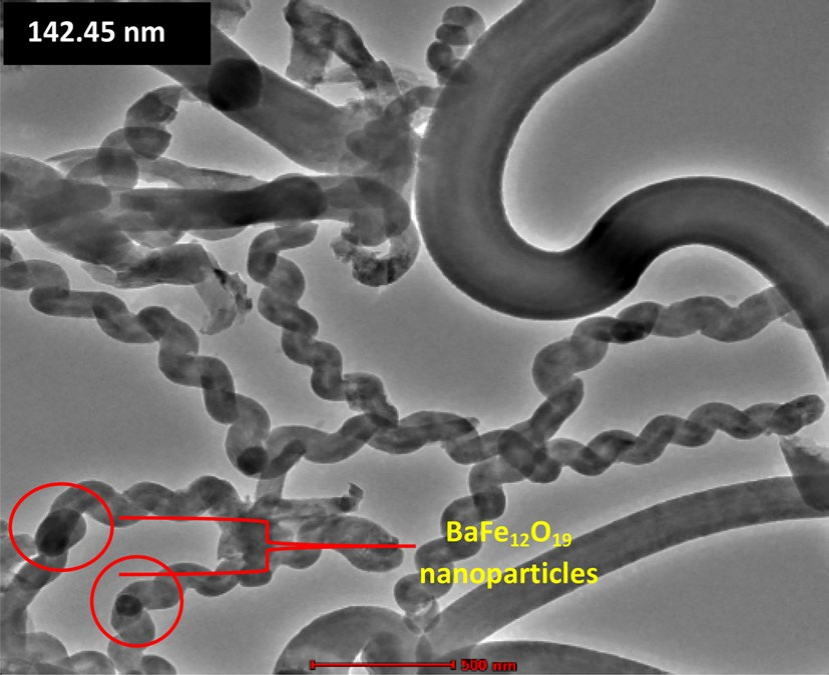
HRTEM image of spiraled MWCNTs/BaFe_12_O_19_ hybrid.

The particles of BaFe_12_O_19_ with an average size of approximately ~ 3.19 µm were homogeneously distributed with agglomeration particles due to the magnetic attraction behaviour (Fig. 3a). The hybridisation of spiraled MWCNTs/BaFe_12_O_19_ after the CVD process showed that the carbon structures were mostly formed in a straight-like, spiral and twisted fibre structures (Fig. 3b). However, we observed net-like fibres which were created by the aggregation of excellent fibres and particle-like carbon, and we also noticed that the fibres were highly aggregated and favoured in spiral coil form. The average outer diameter of the spiraled MWCNTs/BaFe_12_O_19_ hybrid synthesised was approximately ~ 120.74 nm (Fig. 3b). Furthermore, the presence of BaFe_12_O_19_ nanoparticles structures in the enlarged image can be clearly seen in (Fig. 3c). Absorption and dissociation of a carbon precursor on the surface of a catalyst particle and dissolution of carbon into the catalyst particle are the commonly suggested mechanisms for carbon fibre growth. The carbon crystallises the metal particle after the catalyst particle is loaded with carbon and extruded to form CNTs or CNF^12,13^. CNTs are commonly found as cylinders of rolled-up graphene sheets^14^, resulting in single-walled, double-walled, and multi-walled entities. Coiled tubes occur in one of the two types of helical materials where an inner hollow occurs along the length of the coil^15^. Both straight, spiral fibres and dark spot which BaFe_12_O_19_ particles can be seen from the HRTEM image have an average outer diameter of approximately ~ 142.45 nm (Fig. 4). The presence of hollow and tube-like structures confirms the tubular nature structure of MWCNTs (Fig. 4), and we believe that the coil growth process involves a core cluster formation followed by a helical tube formation. As shown in the FESEM and HRTEM morphology images (Figs. 3b, c, 4), the BaFe_12_O_19_ nanoparticles are strongly attached to the surface and tips of MWCNTs. The HRTEM morphology image displayed the conductive MWCNTs pathways formed by an interconnected network of spiraled MWCNTs/BaFe_12_O_19_ hybrid embedded in the composites resin matrix. The network structure of a nanocomposite with a large surface area is expected to result in a variety of interfacial polarisation in the hybrid nanocomposite. The difference in electrical conductivity between spiraled MWCNTs and BaFe_12_O_19_ nanoparticles also create high interfacial polarization in the hybrid nanocomposite. The interfacial polarisation occurs when the motion of moving charge is impeded at the interfaces of a material. There could be multiple interfacial polarisation due to the large specific areas of both MWCNTs and BaFe_12_O_19_ nanoparticles, which can lead to an increase in complex dielectric permittivity values (Fig. 9). Based on the previous article, a researcher reported on the detailed mechanism of the reaction^28^.

Most of the synthesised MWCNTs fibres show a spiraled structure. A regularly orientated nucleation of pentagonal, hexagonal and heptagonal carbon rings along the nanotube body plays essential roles in producing a coiled nanotube, and details of its growth mechanisms have been proposed and described previously^16,17^. This spiral structure brings advantages in attenuating and EM wave within the material as reported by^18^. On the other hand, short fibre structure improves the performance of EM wave absorption as compared to microparticles^19^. It is due to their properties of high surface-to-volume ratio, quantum size effects and the network structure effect of the material in the composite^3^. The black spots in HRTEM image are the catalyst from BaFe_12_O_19_ due to higher atomic number compared to CNTs which give black contrast to the image.

### Raman analysis

**Figure 5:**
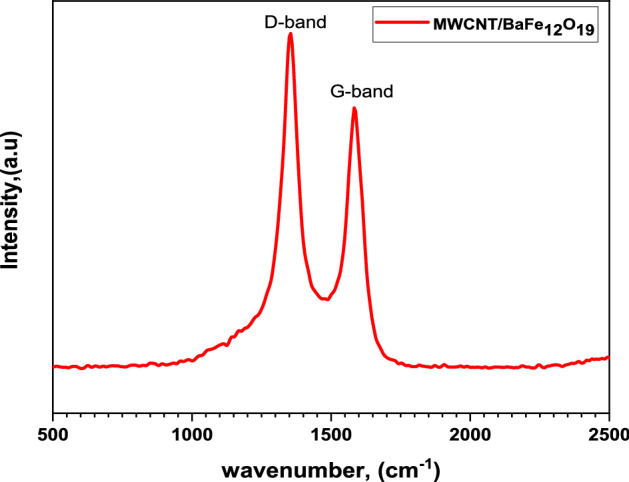
Raman spectra of spiraled MWCNTs/BaFe_12_O_19_ hybrid.

The presence of spiraled MWCNTs/BaFe_12_O_19_ hybrid is further confirmed by analysing the Raman spectrum in a frequency range of 500–2500 cm^−1^ (Fig. 5). The stretching of sp^2^ hybridised carbon in MWCNTs is reflected in two influential bands which appeared at around 1350 and 1600 cm^−1^ as the defect (D) band and graphite (G) band respectively. The ratio of the intensity of G- to D-bands in the spectrum of spiraled MWCNTs/BaFe_12_O_19_ hybrid is ∼ 0.74 that shows many defects are formed with higher intensities compared to the graphite peak. It has been reported that the defects in CNTs structure can act as polarisation centres and may contribute to strong microwave absorption^20^. Since the composite samples prepared were spiraled MWCNTs, they are found to have more defects due to their complicated structures, and this indicates better microwave absorption which was mainly attributed to the dielectric relaxation^20^.

### Elemental analysis

**Figure 6:**
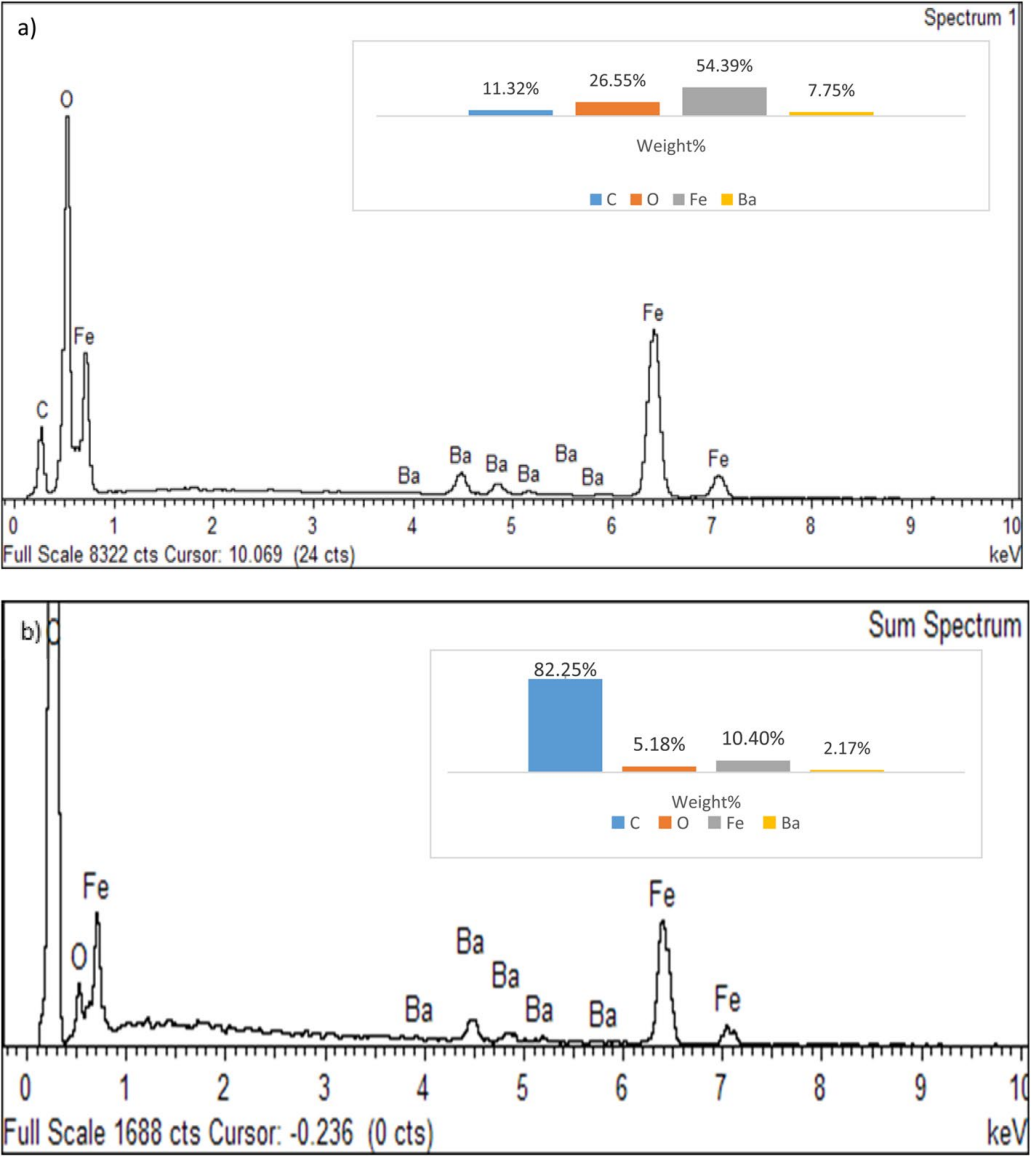
EDX pattern of (**a**) barium hexaferrite (BaFe_12_O_19_) and (**b**) spiraled MWCNTs/BaFe_12_O_19_ hybrid.

**Figure 7:**
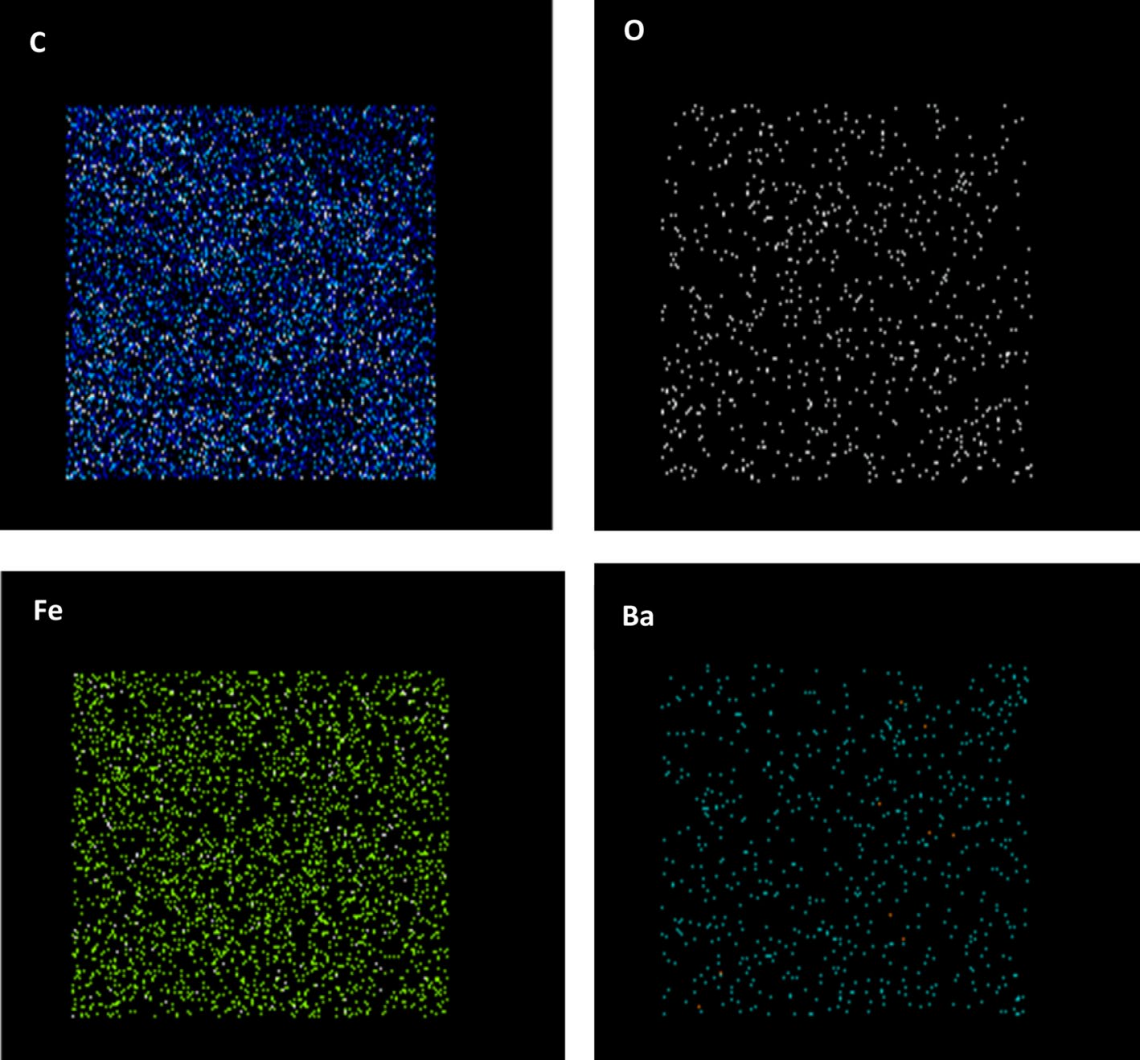
EDX an elemental mapping figures distribution of spiraled MWCNTs/BaFe_12_O_19_ hybrid.

Quantitative elemental analysis of BaFe_12_O_19_ and spiraled MWCNTs/BaFe_12_O_19_ hybrid was employed using energy-dispersive X-ray (EDX) to confirm the chemical composition in those samples (Fig. 6a, b). The EDX spectra of BaFe_12_O_19_ nanoparticles revealed the presence of oxygen (O), barium (Ba), iron (Fe) and carbon (C) peak. The appearance of carbon (C) peak may be due to the carbon tape from the preparation of a sample for characterisation (Fig. 6a). The analysis result from EDX shows that the average contents of Ba, Fe, O and C elements in powders are about 7.75%, 54.39%, 26.55% and 11.32% respectively. Meanwhile, the EDX spectra of spiraled MWCNTs/BaFe_12_O_19_ hybrid also revealed the existence of oxygen (O), barium (Ba), iron (Fe) and carbon (C) peaks with no other contaminated constituent as perceived in Fig. 6b. The analysis result from EDX shows that the average contents of Ba, Fe, O and C elements in powders are about 2.17%, 10.4%, 5.18% and 82.25% respectively. Ba, Fe, O and C elements in spiraled MWCNTs/BaFe_12_O_19_ hybrid were distributed as seen in Fig. 7.

### Magnetic properties

**Figure 8:**
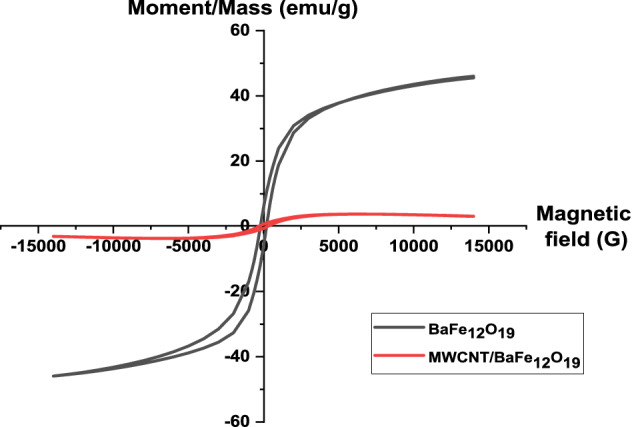
Room temperature hysteresis loop of barium hexaferrite (BaFe_12_O_19_) and spiraled MWCNTs/BaFe_12_O_19_ hybrid.

Figure 8 shows the hysteresis curves for BaFe_12_O_19_ nanoparticles powders and the spiraled MWCNTs/BaFe_12_O_19_ hybrid. The ferromagnetic trend with a very small saturation magnetisation could be observed in the hysteresis loop of MWCNTs^21^. The hysteresis loops show that the values of saturation magnetisation (*Ms*) of spiraled MWCNTs/BaFe_12_O_19_ hybrid are lower than the BaFe_12_O_19_ nanoparticles sample (Table 1). This is due to the presence of MWCNTs as a phase with a very low saturation magnetisation structural distortion in the surface of hexaferrite nanoparticles caused by the interaction of the metal ions transition with the oxygen atoms in the magnetoplumbite structure and strain between hexaferrite nanoparticles and MWCNTs^21–23^. As for coercive force (*H*_*c*_), the value increased for the spiraled MWCNTs/BaFe_12_O_19_ hybrid compared to BaFe_12_O_19_ nanoparticles (Table 1). This could also be related to the improvement of some of the surface homogenities of ferrite nanoparticles and the surface pinning of the magnetic moment. The surface magnetic anisotropy of nanoparticles could be increased in the interface of CNTs and nanoparticles that may lead to the increase of coercivity.

**Table 1:** Saturation magnetisation, *Ms* and coercivity, *Hc* value for barium hexaferrite (BaFe_12_O_19_) and spiraled MWCNTs/BaFe_12_O_19_ hybrid.

Sample code	Saturation magnetisation, *Ms* (emu/g)	Coercivity, *Hc* (Gauss)
BaFe_12_O_19_	45.10	202.94
MWCNTs/BaFe_12_O_19_	3.75	293.64

### Microwave characteristics

**Figure 9:**
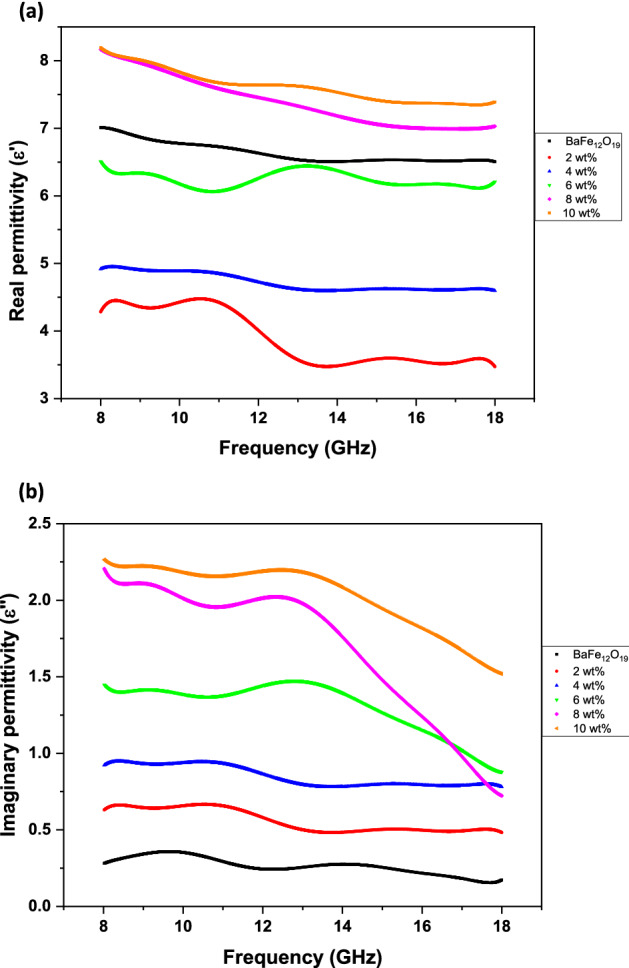
Frequency dependency of (**a**) real and (**b**) imaginary parts of the complex permittivity (*ε*_*r*_) values for BaFe_12_O_19_ and spiraled MWCNTs/BaFe_12_O_19_ hybrid with different filler contents (2, 4, 6, 8 and 10 wt%) and with thicknesses of *d* = 2 mm.

**Figure 10:**
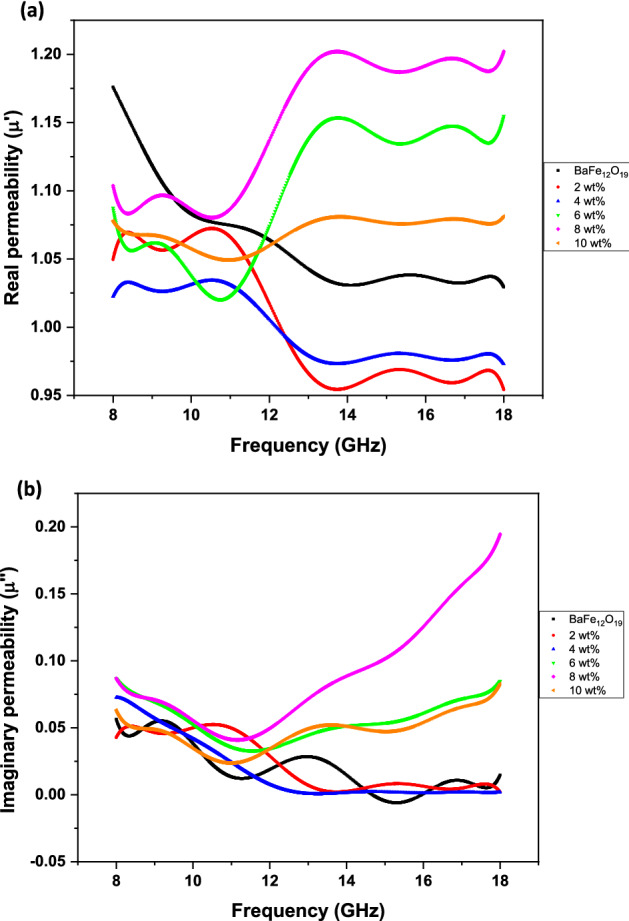
Frequency dependency of (**a**) real and (**b**) imaginary parts of the complex permeability (*μ*_*r*_) values for BaFe_12_O_19_ and spiraled MWCNTs/BaFe_12_O_19_ hybrid with different filler contents (2,4, 6, 8 and 10 wt%) and with thicknesses of *d* = 2 mm.

**Figure 11:**
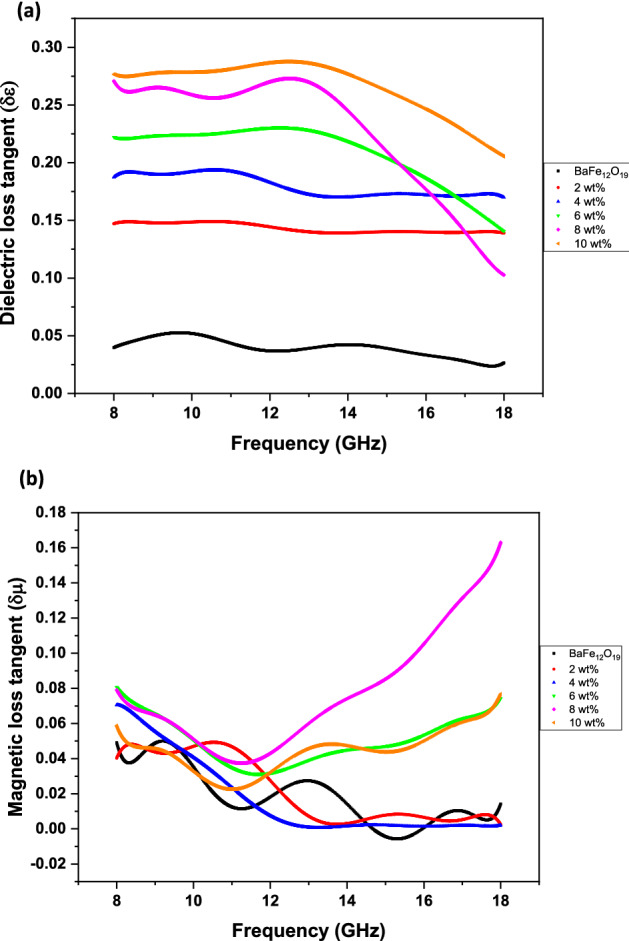
Frequency dependency of (**a**) dielectric loss tangent (tan *δɛ*) and (**b**) magnetic loss tangent (tan *δµ*) values for BaFe_12_O_19_ and spiraled MWCNTs/BaFe_12_O_19_ hybrid with different filler contents (2, 4, 6, 8 and 10 wt%) and with thicknesses of *d* = 2 mm.

**Figure 12:**
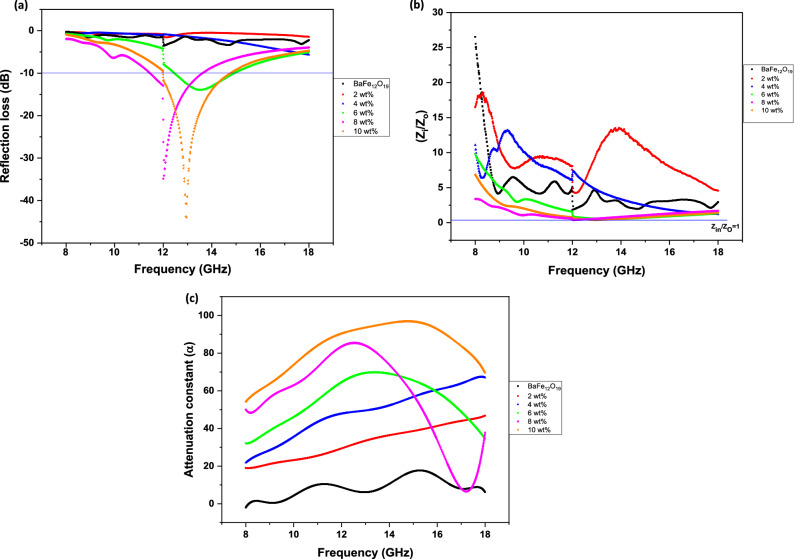
Frequency dependency of (**a**) reflection loss (*RL*), (**b**) impedance matching and (**c**) attenuation constant values for BaFe_12_O_19_ and spiraled MWCNTs/BaFe_12_O_19_ hybrid with different filler contents (2, 4, 6, 8 and 10 wt%) and with thicknesses of *d* = 2 mm.

The dielectric and magnetic properties of the composites were investigated by measuring the complex permittivity (*ɛr* = *ɛ′ − jɛ″*) and the complex permeability (*μr* = *μ′ − jμ″*) in a frequency range of 8–18 GHz. The variation of real (*ε′*) and imaginary (*ε″*) parts of complex permittivity (*ɛ*_*r*_) with a frequency of BaFe_12_O_19_ and spiraled MWCNTs/BaFe_12_O_19_ hybrid with different filler contents are presented in Fig. 9. The value of complex permittivity is higher at low frequencies and lowers down at high frequencies for all samples. These behaviours could be explained by electronic and ionic polarisation considerations, intrinsic electric dipole polarisation and electron hoping^24^. As expected, the imaginary (*ε″*) parts of complex permittivity of barium hexaferrite sample could significantly be improved by adding spiraled MWCNTs (Fig. 9b)^25^. Generally, the complex permittivity (*ε*_*r*_) values of spiraled MWCNTs/BaFe_12_O_19_ hybrid decrease and remain almost constant with the increase of frequency, which is in agreement with the regular rules of polarisation relaxation^26^. The polarisation of the inner electric dipole in dielectrics was unable to keep pace with the change of frequency. Due to the rising of the frequency, the polarisation weakened, and the complex permittivity (*ε*_*r*_) values diminished^27^. Therefore, a significant enhancement is achieved in both *ε′* and *ε″* with the increasing filler content of spiraled MWCNTs/BaFe_12_O_19_ hybrid loading, ranging from 2 to 10 wt%. The enhancement of *ε*_*r*_ further confirms the shift of obtained *RL* peak as shown in Fig. 12a for as-prepared composites. Prior to the 10 wt%, the increment of *εr* may be attributed to the enhanced dipole polarisation, interfacial polarisation or both^27^. The frequency dependency of real (*μ′*) and imaginary (*μ″*) parts of the complex permeability (*µ*_*r*_) values for BaFe_12_O_19_ and spiraled MWCNTs/BaFe_12_O_19_ hybrid with different filler contents are presented in Fig. 10. Based on the findings of this study, we suggest that MWCNTs do not have a significant impact on the complex permeability (*µ*_*r*_) of spiraled MWCNTs/BaFe_12_O_19_ hybrid. This is due to an expected behaviour resulted from negligible or a low magnetic property of MWCNTs. However, the values of *μ′* and *µ″* decrease with the increased frequency of samples between 2 to 10 wt%. Generally, the decreasing trend values of *µ′* and *µ″* were attributed to the relaxation of magnetisation induced by domain wall displacement at a lower frequency and spin rotation at an upper frequency in the samples^28^. However, in this experiment, the domain wall contribution did not happen, and it depended on spin relaxation since we were measuring the frequency at between 8 and 18 GHz. The magnetic spin resonance governed the relaxation of BaFe_12_O_19_ as shown by these formulae^29^:

1 $$X_{spin} = { 2}\pi {\text{Ms}}\;{2}/{\text{K}}$$

where *M*_*s*_ is the saturation magnetisation and *K* is the total anisotropy. This is because the size of BaFe_12_O_19_ was made smaller (3.18 µm) due to HEBM process; hence, the domain wall formation does not occur, and only spin rotation plays the role of absorbing the EM wave. Moreover, there is no visible resonance behaviour in the *μ″* spectrums of samples with BaFe_12_O_19_ and spiraled MWCNTs/BaFe_12_O_19_ hybrid (Fig. 10b). The dielectric and magnetic tangent loss of BaFe_12_O_19_ and spiraled MWCNTs/BaFe_12_O_19_ hybrid with different filler contents with a thickness of *d* = 2 mm are presented in Fig. 11. We observed that all samples exhibited much higher tan *δɛ* values than those of tan *δµ* in the whole frequency range, indicating the dielectric loss plays the main role in the EM wave absorption. The spiraled MWCNTs/BaFe_12_O_19_ hybrid exhibits enhanced tan *δɛ* values with increasing filler content while the tan *δµ* values slightly increased, and then dropped at 10 wt% when the spiraled MWCNTs/BaFe_12_O_19_ hybrid content increased with the increasing of the frequency.

Electromagnetic reflection loss (*RL*) of BaFe_12_O_19_ and spiraled MWCNTs/BaFe_12_O_19_ hybrid with different filler wt% and a thickness (*d*) of 2 mm in the 8–18 GHz frequency range is presented in Fig. 12a. The EM wave absorption parameters of the prepared samples are tabulated in Table 2. According to the transmission line theory, a general expression for normalised input impedance, *Z*_*in*_, of the metal-backed microwave absorption layer is^30^:

2 $$Z{\text{in}} = Z_{o} \left( {\frac{{\mu {\text{r}}}}{{\varepsilon {\text{r}}}}} \right)^{1/2} {\text{tanh}}\left( {j\frac{{2\pi fd\left( {\frac{{\mu {\text{r}}}}{{\varepsilon {\text{r}}}}} \right)^{1/2} }}{c}} \right)$$

**Table 2:** Electromagnetic microwave absorption parameters of prepared composite samples.

Sample ratio (wt%)	Thickness of sample, *d* (mm)	Resonance frequency*, f*_*m*_(GHz)	Minimum Reflection loss, *RL* value (dB)	Bandwidth GHz (*RL* < − 10 dB)
BaFe_12_O_19 (_without MWCNT)	2	12.00	− 3.58	–
2		12.09	− 1.54	–
4		18.00	− 5.68	–
6		13.53	− 13.88	2.46
8		12.00	− 34.85	1.97
10		12.96	− 43.99	2.56

where *Z*_0_ = (*μ*_0_*/ε*_0_)^1/2^ is the impedance of vacuum, *c* is the velocity of light in free space, *d* is the thickness of the absorber, *f* is the frequency of the electromagnetic wave while *ɛ*_r_ and *µ*_r_ are the complex permittivity and complex permeability of the composite medium. The impedance matching condition representing the perfect absorbing properties is given by *Z*_*in*_*/Z*_*o*_ = 1.

The reflection loss (*RL*) of the electromagnetic radiation for a single microwave absorbing layer can be expressed as shown in Eq. 3^10,31^.

3 $$RL \left( {dB} \right) = 20 \log \left| {\frac{Zin - Zo}{{Zin + Zo}}} \right|$$

When the characteristic impedance of free space is matched with the input characteristic impedance of an absorber; where *Z*_*in*_ = *Z*_*o*_, the impedance matching condition may occur. In addition, electromagnetic energy can be absorbed completely and dissipated into heat through magnetic and dielectric losses. *RL* results of BaFe_12_O_19_ and spiraled MWCNTs/BaFe_12_O_19_ hybrid with different filler content (wt%) composites are described in Fig. 12a. The calculation of the reflection loss was made for composites thickness (*d*) of 2 mm. The maximum reflection loss (*RL*) (~ − 43.99 dB) and the bandwidth was observed to cover 2.56 GHz for the sample with 10 wt% and a matching frequency of 12.96 GHz (Fig. 12a). We also observed that the *RL* value increases with the increasing filler content (wt%) of spiraled MWCNTs/BaFe_12_O_19_ hybrid, ranging from *RL* = − 1.54 dB, − 5.68 dB, − 13.88 dB, − 34.85 dB and − 43.99 dB with filler content (wt%) = 2,4,6,8 and 10 respectively. Those *RL*s are higher than that of BaFe_12_O_19_ sample without MWCNTs (maximum *RL* = − 3.58 dB at 2 mm thickness) in the measuring frequency range as shown in Table 2. This indicates that the hybridisation of spiraled MWCNTs/BaFe_12_O_19_ hybrid helps in enhancing the EM wave performance.

As the filler content (wt%) of spiraled MWCNTs/BaFe_12_O_19_ hybrid increases, the MWCNTs spontaneously form larger aggregates and agglomerates. Meanwhile, the permittivity of the spiraled MWCNTs/BaFe_12_O_19_ hybrid samples increases as the MWCNTs wt% filler content increases. It can be seen that the imaginary part (energy loss) of permittivity from (Fig. 9b) and dielectric loss tangent from (Fig. 11a) of spiraled MWCNTs/BaFe_12_O_19_ hybrid with MWCNTs loadings from 2 to 10 wt% are substantially increased, and this also shows the frequency-dependence that resembles the absorption ratio of the spiraled MWCNTs/BaFe_12_O_19_ hybrid. Hence, we conclude that the primary enhancement of microwave absorption of the spiraled MWCNTs/BaFe_12_O_19_ hybrid samples is due to the dielectric loss of the composites.

The bandwidth *RL* = − 10 dB (The *RL* = − 10 dB) is an indicator of 90% absorption of the EM wave as reported previously^32^. It is also well known that the enhancement of microwave absorption performance can mainly be ascribed to the good impedance matching ratio, high values of tan *δɛ* and tan *δµ*, and good compensation between the dielectric loss and magnetic tangent loss^33^. Based on our results, we found that the enhancement of microwave absorption abilities of the spiraled MWCNTs/BaFe_12_O_19_ hybrid (10 wt%) composite has resulted in a maximum absorption loss of *RL* − 43.99 dB with a bandwidth of 2.56 GHz at a frequency of 12.96 GHz. The microwave absorption of the sample with a filler content of 10 wt% has been perceived to be increasing due to a suitable matching between the magnetic loss and dielectric loss with a strong attenuation characteristic^10^. The enhancement of microwave absorption properties of the spiraled MWCNTs/BaFe_12_O_19_ hybrid originated from the good impedance matching between the magnetic and dielectric losses parameter^34^.

Besides, a normalised impedance of spiraled MWCNTs/BaFe_12_O_19_ hybrid sample with 10 wt% filler content in the frequency range from 12 to 14 GHz is approximate to 1 (Fig. 12b). Meanwhile, a normalised impedance of sample with a filler content of 6 wt% and 8 wt% in the frequency range from 12 to 15 GHz and 11 GHz and 13.50 GHz respectively shows a nearly matched impedance which is approximately equal to 1 within their frequency range.

To further evaluate the microwave absorption properties of the spiraled MWCNTs/BaFe_12_O_19_ hybrid, the attenuation constant (*α*) is introduced^35^:

4 $$\alpha = \frac{\surd 2\pi f}{c} \times \sqrt {\left( {\mu^{\prime\prime}\varepsilon^{\prime\prime} - \mu^{\prime}\varepsilon^{\prime}} \right) + \sqrt {\left( {\mu^{\prime\prime}\varepsilon^{\prime\prime} - \mu^{\prime}\varepsilon^{\prime}} \right){2 } + \left( {\mu^{\prime}\varepsilon^{\prime\prime} + \mu^{\prime\prime}\varepsilon^{\prime}} \right){2 } } }$$

The attenuation constants of the samples versus frequency are shown in Fig. 12c. The attenuation constants of all hybrid sample are higher than that of pure BaFe_12_O_19_. Therefore, the hybrid samples exhibit higher microwave absorption performance due to their optimal impedance matching and larger attenuation constant. As predicted, the sample with a filler content of 10 wt% has the highest value of attenuation constant compared to other samples. Therefore, this sample is the best candidate for the electromagnetic wave absorption compared to others because of its excellent impedance matching features with the largest value of attenuation constant.

**Figure 13:**
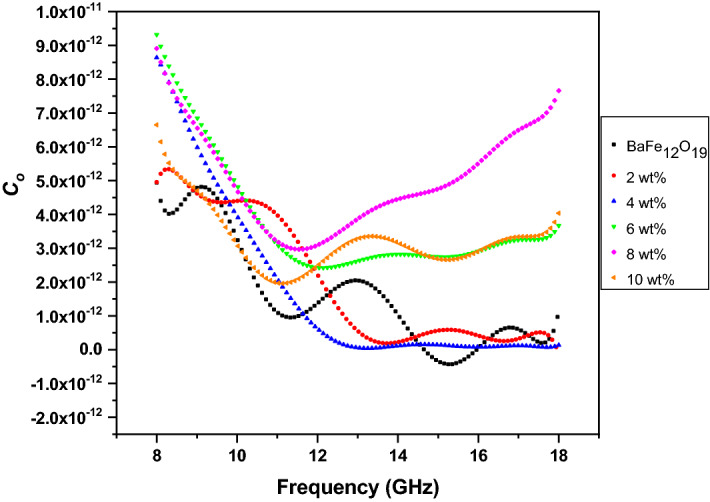
*C*_*o*_ versus frequency of BaFe_12_O_19_ and spiraled MWCNTs/BaFe_12_O_19_ hybrid with different filler content (2, 4, 6, 8 and 10 wt%) and with thicknesses of *d* = 2 mm.

Generally, the magnetic loss originated from the domain wall resonance and natural resonance^36^. At microwave frequencies, eddy current and ferromagnetic resonance are factors that contribute to magnetic loss. The magnetic loss comes only from the eddy current when *C*_*o*_ is constant in the considered frequency range. The effect of eddy current coefficient (*μ*″(*μ′*)^*−*2*f.*−1^) is investigated by introducing the parameter *C*_*o*_ = (*μ*″(*μ′*)^*−*2*f.−*1^)^36,37^. The values of *C*_*o*_ versus frequency are shown in Fig. 13. The values of the quantity for all the samples fluctuated quite distinctly with frequency, except 4 wt%. These results indicated there is just a small contribution from the eddy current loss and it does not play a major role in the magnetic loss within this range of frequency. The *C*_*o*_ for the 4 wt% samples decreased with frequency and showed a more stable behaviour beyond about 13 GHz, ascribing the phenomenon of eddy current effect. Therefore, that the resonance in all of the samples were principally due to natural ferromagnetic resonance within the frequency range. According to previous literature^38,39^, the eddy current loss contribution to the imaginary part of permeability is related to the thickness (*d*) and the electric conductivity (*σ*) of the composite: *μ*″ = 2*πμ*_*o*_(*μ′*)^2^*d*^2^*fσ* where *μ*_*o*_ is the permeability of vacuum. If the magnetic loss only results from the eddy current loss, the values of (*μ*″*μ′*)^*−*2*f.−*1^ should be constant when frequency is varied^40^.

**Figure 14:**
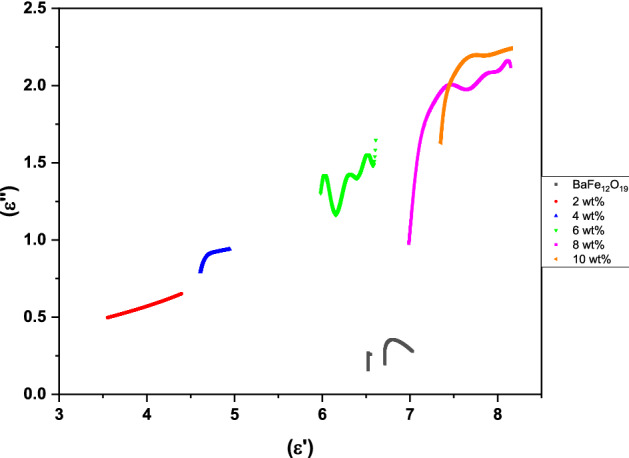
Dielectric Cole–Cole semicircle of the BaFe_12_O_19_ and spiraled MWCNTs/BaFe_12_O_19_ hybrid with different filler content (2, 4, 6, 8 and 10 wt%) and with thicknesses of *d* = 2 mm.

Single semicircle (Cole–Cole semicircle) represents the presence of Debye relaxation (Fig. 14); in particular, a distorted semicircle is due to the combined effect of other loss mechanisms^41^. According to the characteristics of the curves, microwaves have multiple relaxations in BaFe_12_O_19_ and spiraled MWCNTs/BaFe_12_O_19_ hybrid. The result in Fig. 14 shows that the dielectric relaxation of BaFe_12_O_19_ and spiraled MWCNTs/BaFe_12_O_19_ hybrid samples could serve as the centre of polarisation and could provide dipole polarisation.

As summary, the mechanism of this microwave absorption can be ascribed to MWCNTs network. MWCNTs bear more defects and have more degrees of functionalisation. This defect structure, which has multiple bonding linkages, causes interfacial electric polarisation and thus energy dissipation in an alternating electromagnetic field by generating continuous current losses. Next, due to the discontinuity of energy states for the BaFe_12_O_19_ nanoparticles on the surface of MWCNTs, the well-known quantum confinement effect allows electrons to hop from a lower energy state to a higher energy state and hence increasing microwave absorption. Then, for improved microwave performance is also from a suitable combination of epoxy, BaFe_12_O_19_ nanoparticles and MWCNTs concentration with an adequate thickness which forms a multiple scattering network.

## Conclusion

BaFe_12_O_19_ and spiraled MWCNTs/BaFe_12_O_19_ hybrid have been successfully prepared and synthesised via high energy ball milling (HEBM) and via chemical vapour deposition (CVD) technique, respectively. The XRD patterns confirmed the formation of single-phase BaFe_12_O_19_ and the existence of peak carbon of spiraled MWCNTs/BaFe_12_O_19_ hybrid. The morphological study of FESEM and HRTEM revealed that the spiraled MWCNTs structure was formed after the CVD process. The imaginary parts values of the permittivity of BaFe_12_O_19_ (~ 0.1–0.5) and spiraled MWCNTs/BaFe_12_O_19_ hybrid were much higher than those of the pure BaFe_12_O_19_ samples without MWCNTs. Dielectric losses are the main contributors to the microwave absorption properties of the spiraled MWCNTs/BaFe_12_O_19_ hybrid with a filler content of 10 wt%. Good attenuation constant properties were exhibited by samples with a filler content of 10 wt% of spiraled MWCNTs/BaFe_12_O_19_ hybrid. Eddy current effect was shown to be the main contributor of the magnetic losses of spiraled MWCNTs/BaFe_12_O_19_ hybrid. The *RL* peak exhibited a maximum loss of − 43.99 dB at a frequency of 12.96 GHz with a bandwidth of 2.56 GHz for losses less than − 10 dB at the thickness of 2 mm with a filler content of 10 wt%, and this value also corresponds to 99.99% of the EM wave being absorbed by the materials. The result also showed good compatibility of dielectric and magnetic properties in the sample. As a result, spiraled MWCNTs/BaFe_12_O_19_ hybrid samples have the characteristics of a suitable candidate for microwave absorber applications. Also, the *RL* was dependent on the absorber spiraled MWCNTs/BaFe_12_O_19_ hybrid with a different filler content wt%.
